# Insights into Myopia from Mouse Models

**DOI:** 10.1146/annurev-vision-102122-102059

**Published:** 2024-09-02

**Authors:** Reece Mazade, Teele Palumaa, Machelle T. Pardue

**Affiliations:** 1 Department of Ophthalmology, Emory University School of Medicine, Atlanta, Georgia, USA; 2 Institute of Genomics, University of Tartu, Tartu, Estonia; 3 Eye Clinic, East Tallinn Central Hospital, Tallinn, Estonia; 4 Center for Visual and Neurocognitive Rehabilitation, Atlanta VA Healthcare System, Atlanta,Georgia, USA

**Keywords:** axial length, emmetropization, eye growth, mouse model, myopia, refractive error

## Abstract

Animal models are critical for understanding the initiation and progression of myopia, a refractive condition that causes blurred distance vision. The prevalence of myopia is rapidly increasing worldwide, and myopia increases the risk of developing potentially blinding diseases. Current pharmacological, optical, and environmental interventions attenuate myopia progression in children, but it is still unclear how this occurs or how these interventions can be improved to increase their protective effects. To optimize myopia interventions, directed mechanistic studies are needed. The mouse model is well-suited to these studies because of its well-characterized visual system and the genetic experimental tools available, which can be combined with pharmacological and environmental manipulations for powerful investigations of causation. This review describes aspects of the mouse visual system that support its use as a myopia model and presents genetic, pharmacological, and environmental studies that significantly contribute to our understanding of the mechanisms that underlie myopigenesis.

## 1. INVESTIGATING MECHANISMS IN ANIMAL MODELS TO UNDERSTAND MYOPIGENESIS

Animal models are invaluable in furthering our understanding of myopia, a condition of refractive development in which the eye often grows too long for its optical power, causing blurred distance vision. There has been extensive effort to understand how disrupted visual input alters eye growth. Over the past 45 years, numerous animal models have been adapted for studying refractive development, including macaques, chickens, tree shrews, guinea pigs, marmosets, fish, and mice (for a review, see Troilo et al. 2019). While our knowledge of several components of refractive development and myopia is improving, we still lack a complete synthesis of the mechanisms that initiate, drive, and sustain myopigenesis. Understanding this synthesis is critical, as the rates of myopia are rapidly increasing to a projected prevalence of 50% of the global population by the year 2050 (Holden et al. 2016). These numbers are astounding, and myopia will soon become the most common visual health concern, one that increases the risk of developing potentially blinding diseases such as myopic macular degeneration, retinal detachment, glaucoma, and cataract (Haarman et al. 2020). Unsurprisingly, the global cost of myopia or related complications is estimated in the hundreds of billions of dollars per year (Naidoo et al. 2019).

Currently, the primary interventions used by clinicians and optometrists for myopia control in children include the application of pharmaceutical eye drops, such as atropine, a nonspecific muscarinic acetylcholine receptor antagonist, and optical devices (i.e., spectacles or contact lenses of different defocus parameters) (for reviews, see Jonas et al. 2021, Wildsoet et al. 2019). Both can be quite effective at slowing myopia progression, but despite their success, the underlying mechanisms behind myopia inhibition are still unclear. Additionally, environmental control, which consists of changing the visual environment (e.g., increasing bright light exposure) or limiting exposure to myopigenic environments (i.e., indoors), is also gaining in popularity, particularly in countries with high rates of prevalence. However, these strategies slow but do not stop or reverse myopia, and children still spend significant time in myopigenic indoor environments.

To tackle the increasing prevalence of myopia, and to optimize myopia interventions, targeted mechanistic studies are desperately needed. Among animal models, the mouse is particularly well-suited to fill these gaps in knowledge. Its use as a model system for studying myopia has increased significantly over the past decade, with the number of published manuscripts doubling in the past five years. This is largely due to the experimental tools available to overcome limitations in studying the small mouse eye, the extensively characterized mouse visual system, and the ability to manipulate mouse genetics. Additionally, using the mouse allows one to tackle hypotheses from three critical angles, genetics, pharmacology, and environment, while also allowing for the combination of these approaches for more robust investigations. In this article, we discuss why the mouse is especially useful for studying refractive development and review genetic, pharmacological, and environmental mouse studies that have significantly contributed to our understanding of the mechanisms that underlie myopigenesis. We focus on peer-reviewed publications that incorporate a manipulation or intervention to assess mechanisms of refractive eye growth and report refractive error and/or axial length (AL) when measuring refractive development or validating a mouse model of myopia.

## 2. THE MOUSE AS A MODEL FOR REFRACTIVE DEVELOPMENT

Emmetropization is the developmental process of adjusting the eye’s optics and size so that light focuses on the retina. Young children are hyperopic (i.e., far-sighted) such that light focuses behind the retina [plus diopter (D) defocus], although accommodation, i.e., the ability to change the shape of the lens to focus on close objects, can compensate for these small amounts of hyperopia. As the eye grows during development, refractive error moves toward emmetropia, achieving clear vision with light focusing on the retina (0 D). When this process fails, the eye continues to grow, surpassing emmetropia and becoming myopic (minus D defocus). Although the mechanisms underlying emmetropization, and the failure to emmetropize, are unknown, the consensus is that defocused light sensed by the retina is translated to signals in the sclera to regulate axial elongation. When eye size matches optical focus, such that there is no relevant defocus, the system should instruct growth to stop. If this is true, then visual input is critical for refractive development and myopia.

In support of this idea, animals require light for normal refractive development and to respond to visual defocus for myopia induction (for a review, see Troilo et al. 2019). Moreover, adjusting visual defocus using lenses of different power causes the eye to grow to match the imposed defocus (Schaeffel et al. 1988). While defocus is central to this hypothesis, it is important to note that many visual cues are interdependent. For example, myopigenic environments, such as those indoors, contain very different visual cues than protective outdoor environments, such as reduced ambient luminance, fewer high spatial frequencies, increased blur, and stunted wavelength range (for a review, see Lingham et al. 2020). Therefore, many aspects of visual input could be required for emmetropization. To elucidate the mechanisms underlying refractive and myopic development, a good animal model is required, which should have similar biological and physiological systems to those of humans for the detection and translation of visual input. The mouse mostly fulfills this requirement and provides significant experimental benefits for studying mechanisms of refractive development that could apply to human myopia.

### 2.1. Mice Rely on Their Vision for Important Behaviors

It is commonly thought that mice have poor vision, with the implication that they rely more on other senses for important behaviors, such as spatial navigation. Poor vision often refers to low visual acuity, and indeed, mice have significantly lower visual acuity (equivalent to approximately 20/2,000 Snellen acuity) (Huberman & Niell 2011, Iyer et al. 2013) when compared to nonrodents and animals with foveas, such as humans (20/20 Snellen acuity). The maximum visual acuity in the mouse is approximately 0.5 grating cycles per degree of visual space (cpd) (Prusky et al. 2000), which is 90 times less than that of humans, at approximately 45 cpd (Frisén & Glansholm 1975). The high visual acuity of humans is restricted to the fovea, and visual acuity in the peripheral retina drops to approximately 0.8 cpd, which is much closer to the maximum acuity in the mouse (Frisén & Glansholm 1975, Prusky et al. 2000). Thus, in the periphery, which comprises most of the retinal area in humans and mice and is thought to play a critical role in myopigenesis (Smith et al. 2005, Stone et al. 2006), mice and humans have similar acuity. However, it is important to consider that the small size of the mouse eye reduces sampling of the visual scene because there are fewer photoreceptors per area of visual space (for a review, see Huberman & Niell 2011).

The assumption that vision is not important in mouse behavior, or that mice do not utilize their vision to perform complicated visual behaviors, is misguided. Mouse retinal neurons can respond to a wide range of stimuli (Baden et al. 2016), but this neuronal activity does not necessarily mean that visual experience is relevant to behavior. However, vision is critical for accurate spatial navigation in mice (Buhot et al. 2001), and when mice are exposed to a rapidly expanding dark circle overhead (i.e., a looming stimulus), they reliably freeze or flee (Yilmaz & Meister 2013), a similar reflex to that seen in humans (King et al. 1992). More recently, it was found that mice use their vision, and not other senses, to track and hunt crickets, keeping a cricket in their binocular visual zone until the moment of capture (Hoy et al. 2016, Michaiel et al. 2020). These studies provide support to the idea that vision is ethologically important for mice, even though they have low visual acuity.

**Figure 1:**
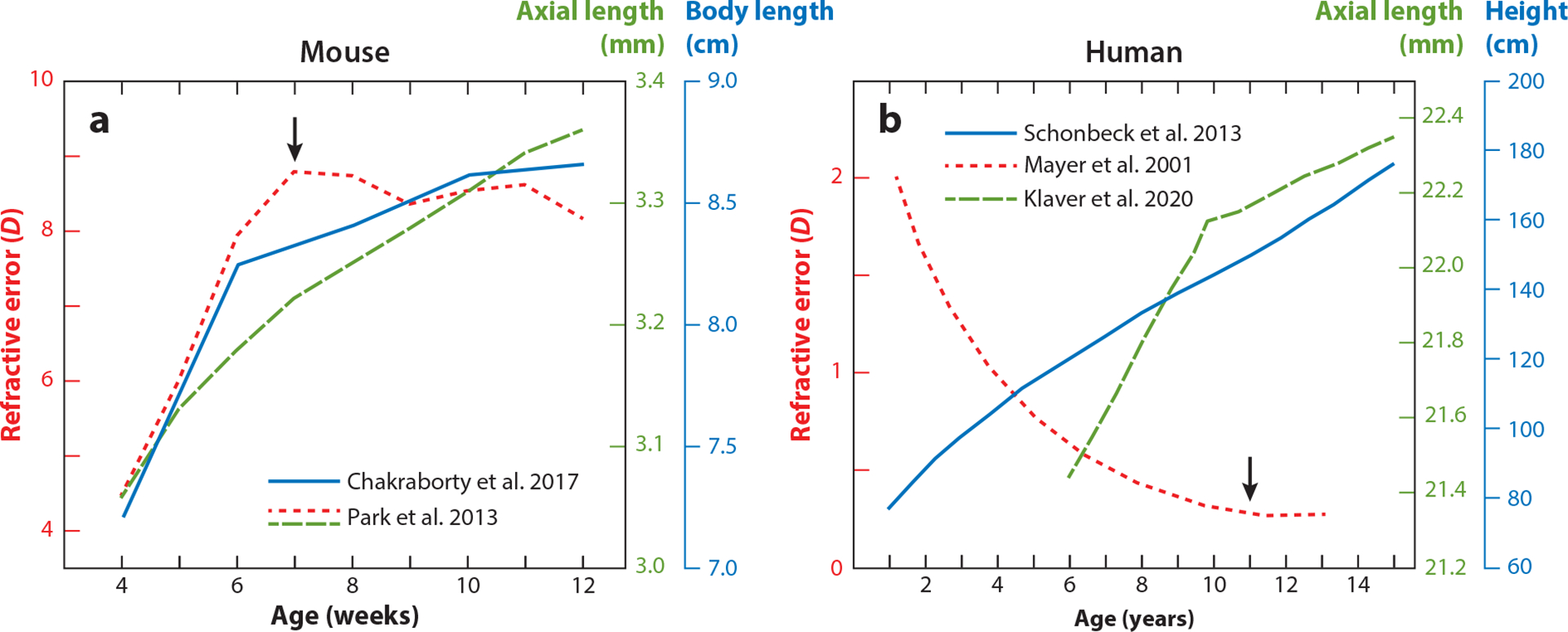
Refractive development plateaus before ocular and body development. Refractive error (*red*) is compared to ocular (*green*) and body (*blue*) growth in (*a*) young mice and (*b*) children. Arrows indicate the age at which refractive error stabilizes. Mouse data taken from Chakraborty et al. (2017) and Park et al. (2013); human data taken from Klaver et al. (2020), Mayer et al. (2001), and Schonbeck et al. (2013).

As in humans, refractive error in mice changes over time to reach a steady state, implying that the optical adjustment optimizes visual signaling. While optical adjustment to a target of 0 D is the goal for humans and other animals (Troilo et al. 2019), it may not be the goal in the typical C57BL/6 mouse. Most studies find that young mice begin hyperopic (approximately 4 D at 4 weeks of age) and become more hyperopic over a few weeks before plateauing at around 7 weeks of age (Figure 1a). Notably, some studies find that mice are myopic at baseline and reach a state closer to 0 D or become slightly hyperopic over time (for a review, see Pardue et al. 2013). Regardless, while refractive error reaches a steady state at around 7 weeks of age, AL and body length, two parameters of overall growth, continue to increase until 12 weeks of age (Chakraborty et al. 2017, Park et al. 2013). Similar trends are seen in humans and suggest that refractive refinement is regulated separately from general growth in both cases (Klaver et al. 2020, Mayer et al. 2001, Schonbeck et al. 2013) (Figure 1b). Moreover, myopia can be reliably induced in mice by manipulation of spatial input. This usually involves covering one eye with either frosted diffusers [form-deprivation myopia (FDM)], which only pass diffuse light, or minus powered lenses [lens-induced myopia (LIM)], which place the focal point of light behind the retina and signal axial elongation during development to bring the image into focus (for reviews, see Pardue et al. 2013, Schaeffel & Feldkaemper 2015). This results in a myopic shift—the treated eye develops relative myopia compared to the untreated, contralateral eye. Therefore, it seems that mice refine their visual optics during emmetropization to a refraction that may be appropriate for their visual behaviors.

### 2.2. Similarity of Mouse Eyes to Human Eyes and Visual Systems

To understand the mouse as a model for human myopia, it is important to consider the structure and composition of the mouse’s eye and visual system. Mouse eyes are located laterally on the head, rather than in the front, providing a 320° horizontal visual field (Scholl et al. 2013) but a significantly smaller binocular zone than that in humans (40° versus 140°) (Heesy 2004). While accommodation is an important feature for human vision, mice do not appear to accommodate (Ott 2006). Instead, mice have a large depth of field (approximately 10 D or more) compared to the <0.5 D depth of field in humans (de la Cera et al. 2006, Marcos et al. 1999, Remtulla & Hallett 1985, Schmucker & Schaeffel 2006). The large depth of field in mice generates the question: Can mice resolve changes in defocus within this range? Data support the idea that mice do detect defocus, as they respond to a wide range of lens defocuses that induce myopia (Jiang et al. 2018). Despite these differences in visual fields and optics, the structure of the mouse eye is similar to that of humans, albeit at a much smaller scale (an AL of approximately 3.3 versus approximately 24.5 mm) (Chakraborty et al. 2017, Howland et al. 2004). Due to the smaller eye, most of the internal components are also smaller; however, the lens is relatively much larger, equating to 62% of the AL compared to just 19% in the human eye (Chakraborty et al. 2017, Waring et al. 2021). Surprisingly, the average retinal thickness is similar: approximately 204 μm in mice versus approximately 198 μm in humans, although human retinal thickness varies greatly with eccentricity (Cheng et al. 2010, Ferguson et al. 2013). Lastly, both humans and mice have collagenous sclerae (Rada et al. 2006), an important similarity because scleral remodeling is crucial for axial elongation in myopia development.

In addition to the different ocular structures, there are several important retinal differences between mice and humans to consider. While mice lack a fovea, and the resulting high visual acuity, there is evidence that they have a visual streak located close to the optic nerve. Indeed, this area has high retinal ganglion cell (RGC) density (approximately 5,851 cells/mm^2^) that decreases with eccentricity (<2,000 cells/mm^2^ at 2.5 mm from optic nerve) (Rodriguez et al. 2014, Salinas-Navarro et al. 2009). This is similar to humans but at a much reduced scale (35,100 cells/mm^2^ near the fovea to <5,000 cells/mm^2^ at 4 mm from fovea) (Curcio & Allen 1990). Although mouse and human RGC densities may be similar in the periphery, the average retinal receptive field size in mice (approximately 6°) is significantly larger than in humans (0.08° at the fovea and approximately 1.7° at 70° from the fovea) (Koehler et al. 2011, Ransom-Hogg & Spillmann 1980). In addition to differences in RGC density, mice have significantly higher photoreceptor density that is similar across the retina, whereas in humans, the all-cone fovea creates asymmetrical rod and cone distributions. This results in a rod-to-cone ratio that is very different for the central retina (mice: 44; humans: 0) but very similar for the peripheral retina (mice: 34; humans: 30) (Volland et al. 2015). Furthermore, while rod photoreceptor peak wavelength sensitivity is similar in mice and humans (approximately 497 nm), cone photoreceptor sensitivity is different. Mice have a UV-sensitive cone (360 nm), whereas humans have short- and long-wavelength-sensitive cones (419 and 558 nm, respectively) (Dartnall et al. 1983, Peirson et al. 2018). Although both species do possess similar medium-wavelength cones (508 nm in mice and 531 nm in humans), mice have a cone opsin gradient with UV opsin enriched in the ventral retina and medium-wavelength-sensitive (M) opsin enriched in the dorsal retina (Applebury et al. 2000). In addition to classical opsins, both mouse and human eyes contain atypical opsins, including Opn3 (encephalopsin), Opn4 (melanopsin), and Opn5 (neuropsin) (Upton et al. 2021). Lastly, all major classes of retinal neurons are present in the mouse to signal a wide range of visual stimuli (Baden et al. 2016).

Mouse ocular structure and visual pathways have many similarities to those of humans that can expand our current understanding of myopia. Both the mouse and human periphery are rod dominated, and both rods and the peripheral retina are thought to be critical to myopia development (Smith et al. 2005, Stone et al. 2006). Additionally, the general structure, composition, and pathways of the mouse peripheral retina are very similar to those of humans. However, mice have low optical quality (de la Cera et al. 2006, Geng et al. 2011), no accommodation, and a large depth of field. Furthermore, the largest challenge with the mouse model is the small eye size, which increases the difficulty of obtaining reliable refractive and AL measurements with the typical methods used for animal models with larger eyes. The creation of the mouse automated photorefractor (Schaeffel et al. 2004) solved this problem and allowed for repeatable refractive error measurements in mice. This technique involves using a camera to capture an infrared brightness profile of the back of the mouse eye before calculating the refractive error based on the slope of the brightness profile. Both precalibration of the brightness profile with trial lenses of different powers and alignment of the camera on the back of the eye while avoiding the optic nerve head region are critical components of this method. Importantly, the small eye artifact, a phenomenon in which light reflected from the back of the eye focuses anterior to the photoreceptors, is still present. This results in a more hyperopic measurement [estimated at approximately plus 37 D (Schmucker & Schaeffel 2004)], and thus, mice may not be functionally hyperopic, as most studies indicate. For AL, the utilization of optical coherence tomography (OCT) has been invaluable in obtaining higher-resolution, whole-eye images, as previous methods relied on lower-resolution techniques. This is especially important because only approximately 6 μm of axial growth equals approximately 1 D of refractive change (Schmucker & Schaeffel 2004). While OCT has allowed for less variable measurements due to higher resolution, the technique is still being optimized, and alignment along the same ocular axis when taking measurements may further reduce variability. Measures of both refractive error and AL using these methods are, in general, reproducible across studies and laboratories (for reviews, see Pardue et al. 2013, Troilo et al. 2019). Due to the importance of rigor in the measurements for the small mouse eye, in Table 1, we highlight the measurements used across studies. In many regards, the myopic mouse is a great model for myopic humans due to its similar ocular structure and visual properties. Because mice are small and easy to handle, genetic investigations can be readily combined with environmental and pharmacological manipulations to powerfully probe underlying mechanisms of myopia.

## 3. INSIGHTS INTO THE MECHANISMS OF MYOPIA DEVELOPMENT FROM MICE

Mouse models have emerged as valuable tools in uncovering the complex genetic and environmental factors contributing to myopia. They not only allow for the investigation of underlying molecular pathways, but also provide a platform for testing potential therapeutic interventions. The ability to manipulate specific genes, environmental conditions, and signaling cascades in a well-controlled manner enables the investigation of myopia mechanisms on systems, cellular, and molecular levels. Mouse myopia studies have based their hypotheses on prior knowledge of human myopia or have focused on understanding the underlying ocular light detection and retinoscleral signaling driving myopia development. Below, we present and discuss numerous studies using mouse myopia models, which are specified explicitly in Table 1 (e.g., FDM or LIM with different negative diopter lenses).

### 3.1. Hypotheses Derived from Human Studies

A significant portion of mouse myopia studies build on prior investigations in humans, which have identified key genetic and environmental risk factors of myopia. Mouse studies have taken advantage of our knowledge of syndromes and other eye diseases associated with myopia, genetic factors modulating the risk of myopia, and the effect of pharmacological agents on eye growth, as well as observations of myopic eyes.

#### 3.1.1. Syndromes and diseases associated with myopia.

The data showing that many retinal dystrophies are associated with high myopia in humans have led to investigations of several retinal dystrophy genes in mice. Mouse models of congenital stationary night blindness type 1 (CSNB1) and rod-dominant retinal degenerations have highlighted the importance of rod and ON cone pathways, which both respond to increments of light, in myopia development. For example, the knockout of *Nyx* (nob mice) and *Gpr179*, both associated with CSNB1 and high myopia in humans, resulted in a larger myopic shift with FDM or LIM, coupled with lower retinal levels of dopamine and its primary metabolite, 3,4-dihydroxyphenylacetic acid (DOPAC) (Pardue et al. 2008, Wilmet et al. 2022). Similarly, mice with retinal degenerations caused by *Pde6b*^*rd1*^ and *Pde6b*^*rd10*^ mutations showed larger myopic shifts in response to myopia induction and lower retinal dopamine turnover (Park et al. 2013). Interestingly, under normal refractive development, *Nyx*^−/−^, *Pde6b*^*rd1/rd1*^, and *Pde6b*^*rd10/rd10*^ mice were all more hyperopic than their wild-type (WT) counterparts (Pardue et al. 2008, Park et al. 2013), suggesting that genetic mutation must be combined with myopigenic stimuli to induce myopia. Furthermore, knockdown of *Irbp*, which results in retinal degeneration, leads to myopic refractions, longer AL (Wisard et al. 2011), and larger lenses (Zhu et al. 2021), while retinal dopamine levels remain unchanged (Markand et al. 2016). These findings highlight the critical links between retinal signaling and dopamine metabolism in myopigenesis.

Several other syndromes characterized by high myopia have guided mouse studies, including Donnai-Barrow syndrome, which is associated with mutations in lipoprotein receptor–related protein 2 (*Lrp2*) or megalin; Knobloch syndrome, which is linked with variants of collagen XVIII; and SLITRK6-associated syndrome. Similar to the myopic phenotype observed in humans, the *Lrp2*^−/−^ (Storm et al. 2014), *Col18a1*^−/−^ (Aikio et al. 2013), and *Slitrk6*^−/−^ mice (Tekin et al. 2013) had longer ALs than WT mice, accompanied by retinal pigment epithelium (RPE) defects in the *Lrp2*^−/−^ mice and anterior segment and vasculature defects in the *Col18a1*^−/−^ mice. Further analysis of the *Lrp2* gene has revealed the importance of the RPE in myopia pathogenesis; namely, RPE-specific knockdown of *Lrp2* results in eye enlargement through mechanisms involving the truncated N terminus of SREBP2 (Mai et al. 2022, Zhang et al. 2022). Furthermore, the *Lrp2* knockout phenotype was rescued by RPE-specific overexpression of bone morphogenetic protein 2 (*Bmp2*) (Mai et al. 2022), a gene associated with myopia in humans (Verhoeven et al. 2013). Consistent with these findings, the downregulation of *Bmp2* in the RPE led to eye enlargement (Mai et al. 2022). Familial adenomatous polyposis is caused by mutations in the adenomatous polyposis coli (*APC*) gene and is modeled using mice carrying a nonsense mutation in the *APC* gene (*APC*^*Min*^ mice). The *APC*^*Min*^ mice had relative myopia compared to WT animals, and interestingly, they had shorter ALs and showed thinning of the crystalline lens (Liu et al. 2015). APC is an essential negative regulator in the Wnt signaling pathway, prompting further studies of Wnt signaling effects on myopigenesis. Indeed, pharmacological inhibition of the Wnt signaling pathway by niclosamide slowed myopia development in the *APC*^*Min*^ mice (Liu et al. 2021). Furthermore, inhibiting the Wnt pathway also reduced myopic shift, while its activation increased myopic shift in FDM (Ma et al. 2014). These animal models have been valuable for understanding the molecular events occurring in myopia pathogenesis. Furthermore, the ability of the genetic knockout models to replicate the myopic phenotype observed in humans offers evidence for mechanistic similarities between mouse and human myopia, reinforcing the validity of utilizing mice as a model for studying myopia.

#### 3.1.2. Genetic risk factors of myopia.

Advancements in genetic analyses have resulted in the discovery of numerous single-nucleotide variants (SNVs) associated with eye growth, prompting further exploration of the genes modulated by these SNVs in mouse models. These genes include Ras protein-specific guanine nucleotide-releasing factor 1 (*RASGRF1*), cathepsin H (*CTSH*), vasoactive intestinal peptide receptor 2 (*VIPR2*), 26S proteasome non-ATPase regulatory subunit 3 (*PSMD3*), zinc finger protein 644 (*ZNF644*), glycine receptor alpha 2 (*GLRA2*), amyloid precursor-like protein 2 (*APLP2*), synthesis of cytochrome C oxidase 2 *(SCO2*), vitamin D receptor (*VDR*), and paired box 6 (*PAX6*) (Table 1). Several mouse models have established a role of these genes in regulating eye growth, as would be expected from our knowledge of human myopia. For example, the *Vipr2*^−/−^ (Zhao et al. 2022) and *Glra2*^−/−^ mice (Tian et al. 2023) were more myopic than WT. Similarly, the *Psmd3* knockin mice (J. Chen et al. 2023) and *Zfp644* mutant mice (Szczerkowska et al. 2019) had significantly increased ALs. In addition, the knockout of *Aplp2*, a gene associated with retinal inhibition through glycinergic amacrine cells, resulted in high degrees of hyperopia and reduced susceptibility to FDM (Tkatchenko et al. 2015). Furthermore, knockdown of the scleral *Vdr* resulted in more myopic refractions, while administering calcipotriol, a vitamin D derivative, reduced myopic shift in FDM (Jiao et al. 2023). *RASGRF1* is located in close proximity to an SNV associated with refractive error. Studies in the mouse, however, showed no effect on the knockdown of *Rasgfr1* on AL, but this gene was associated with a heavier crystalline lens (Hysi et al. 2010). Pathogenic variants of *SCO2* have been proposed to cause high-grade myopia, but a mouse model carrying one such variant had no changes in AL (Piekutowska-Abramczuk et al. 2016). It was also found that eye drop application of anti-miR-328–3p, related to the *PAX6* gene, was as effective as 1% atropine in slowing myopia progression in FDM (Liang et al. 2022). Furthermore, miR-328–3p expression was upregulated by retinoic acid binding to the miR-328-3p promoter (Liang et al. 2022). These results emphasize the usefulness of the mouse model in providing a more thorough understanding of molecular signaling pathways in myopia.

Genetic analyses, coupled with findings from animal studies, have led to the investigation of hypoxia in myopia development. It was shown that genes associated with risk for human myopia are enriched for the hypoxia-inducible factor-1 (HIF-1) signaling pathway (Zhao et al. 2020b), while FDM induced scleral activation of hypoxia signaling pathways and upregulation of HIF-1α (Wu et al. 2018) and HIF-2α (Wu et al. 2022). In mice, scleral HIF-1α and HIF-2α knockdown resulted in hyperopic shift and inhibited the development of FDM (Wu et al. 2022, Zhao et al. 2020b), whereas HIF-1α upregulation resulted in myopia (Zhao et al. 2020b). Further studies have shown that antihypoxic drugs slowed FDM progression (Wu et al. 2018). It has been hypothesized that hypoxia may be causally linked with myopia via the reduction of choroidal blood flow and choroidal thinning during accommodation, which may result in scleral hypoxia (Zhao et al. 2020b). The role of hypoxia in ocular growth has been further explored using the vascular endothelial growth factor (*Vegf*) gene, which is induced by HIF-1α under hypoxic conditions. RPE-specific knockdown of *Vegf* resulted in a thinner choroid and longer AL, while increasing the levels of VEGF by RPE-specific knockout of the von Hippel Lindau factor led to shorter ALs and thicker choroids (Zhang et al. 2022). These findings do not align with the hypothesis that hypoxia results in myopigenesis, as a higher level of VEGF resulted in shorter ALs. This discrepancy was hypothesized to be associated with the role of physiological secretion of VEGF in maintaining the choriocapillaris, which is important in supporting appropriate eye growth (Zhang et al. 2022).

Lastly, several genes associated with microphthalmia (small eyes) have been studied in the mouse, including *PRSS56* and *MFRP*. *Prss56* mutant mice were first discovered in a screen for high intraocular pressure and were also characterized by anterior chamber angle abnormalities. Both the *Mfrp*^−/−^ and *Prss56*^−/−^ mice have shorter ALs than WT mice (Koli et al. 2021, Nair et al. 2011, Paylakhi et al. 2018). Interestingly, the loss of *Prss56* or *Mfrp* in the otherwise myopic *Irbp*^−/−^ mice resulted in normal ALs (Koli et al. 2021). Studies on *Prss56* have highlighted the importance of Müller cells in refractive development. Prss56 was shown to be expressed in a subset of Müller glia in adult retinas, and inducible knockdown of *Prss56* specifically in the Müller cells resulted in axial shortening (Paylakhi et al. 2018). In conclusion, our increasing knowledge of genetic risk factors in myopia has enabled scientists to leverage the powerful genetic tools available in the mouse to better understand the pathogenesis of myopia.

#### 3.1.3. Atropine treatment.

Atropine is an effective treatment for myopia progression in children (Chua et al. 2006). As in humans, atropine slows eye growth in mice, inducing more hyperopic refraction and reducing ALs in normal refractive development, as well as in LIM and FDM (Barathi & Beuerman 2011, Barathi et al. 2014, Jiang et al. 2018). Studies on the potential atropine receptors mediating the inhibitory effect on eye growth led to the discovery that, while normal refractive development was unaltered in the absence of muscarinic acetylcholine receptors (Chrm) 1–5, the *Chrm2*^−/−^ and *Chrm3*^−/−^ mice did not develop a myopic shift in LIM (Barathi et al. 2013). Atropine treatment also effectively reduced the AL of the *Lrp2*^−/−^ mice but did not affect retinal levels of dopamine or DOPAC (van der Sande et al. 2023). These findings suggest that atropine may exert its effect at least partly via Chrm2 or Chrm3 through a mechanism independent of dopamine. Mice have also been used to validate atropine and other candidate US Food and Drug Administration–approved drugs that have been screened for eye growth inhibition (Lin et al. 2021).

#### 3.1.4. Extracellular matrix remodeling of the sclera.

Nearly half a century ago, studies uncovered ultrastructural changes in scleral collagen in highly myopic staphylomatous eyes (Curtin et al. 1979). Follow-up investigations in tree shrews demonstrated lower levels of sulfated glycosaminoglycans in their sclerae upon FDM induction (Norton & Rada 1995), further strengthening the hypothesis that extracellular matrix (ECM) changes in myopic sclerae play a role in myopia pathogenesis. The proteoglycans lumican (Lum) and fibromodulin (Fmod), present in sclerae and other fibrillar collagen-rich connective tissues, have been the focus of several studies. *Lum*^−/−^ *Fmod*^−/−^ animals were shown to have increased ALs and thinner sclerae with abnormal collagen fiber structures (Chakravarti et al. 2003). Importantly, these animals also had cloudy corneas (Chakravarti et al. 2003), and therefore, the myopic phenotype of these animals might result from a combination of *Lum* and *Fmod* mutation and the additional effect of form deprivation from the cloudy cornea. The *Lum* gene was further mapped to chromosome 12q22–23, the MYP3 locus associated with myopia in humans (Farbrother et al. 2004, Young et al. 1998). Using mutant mice carrying the *Lum* mutation identified in humans showed that *Lum*^*L199P*^ mutants replicated the human phenotype, as they had longer ALs. Furthermore, they had abnormalities in their scleral collagen fibers (Song et al. 2016).

Collagen synthesis and ECM production in tissues are modulated by the adenosine A2a receptor (ADORA2a), prompting studies of its effect on myopia. *Adora2a*^−/−^ mice were shown to develop relative myopia and denser scleral collagen fibers compared to their WT littermates (Zhou et al. 2010b). Collagen homeostasis is associated with cAMP signaling, resulting in investigations of the role of phosphodiesterase 4B (PDE4b). It was found that *Pde4b*^−/−^ mice were more myopic than their WT counterparts (Zhao et al. 2021). One of the proteinases mediating collagen breakdown in tissues is matrix metalloproteinase 2 (MMP2). *Mmp2* overexpression via subtenon-delivered adeno-associated virus (AAV) led to myopia in WT mice, while RNA interference–mediated knockdown of *Mmp2* attenuated FDM development (Zhao et al. 2018). Furthermore, fibroblast-specific *Mmp2* deletion resulted in myopic refraction, shorter ALs, and smaller myopic shifts in response to form deprivation (Zhao et al. 2018). Macrophage-specific knockout of *Mmp2* resulted in a smaller myopic shift in FDM (Zhao et al. 2020a). These results highlight the role of scleral MMP2, originating in part from scleral fibroblasts and macrophages, in promoting myopia development.

Scleral remodeling has also been associated with an inflammatory response. LIM can cause endoplasmic reticulum (ER) stress in the sclera, and LIM-induced scleral collagen fiber thinning was blocked with different drugs reducing ER stress (Ikeda et al. 2022). Additionally, knockout of *Nrlp3*, an intracellular sensor detecting pathogens and danger signals, led to less myopic shift and lower levels of inflammatory cytokines in the sclera (Z. Chen et al. 2023). Collectively, these findings provide strong evidence of inflammatory responses contributing to scleral remodeling during myopia development. Moreover, these findings led to investigations of various anti-inflammatory agents in myopia. For example, administration of lactoferrin (Ikeda et al. 2020, Liang et al. 2023), ginkgo biloba extract (Hou et al. 2023), and ω-3 polyunsaturated fatty acids (Hou et al. 2023, Mori et al. 2022, Pan et al. 2021) all suppress myopia development in the mouse model, providing interesting avenues for myopia treatment strategies.

### 3.2. The Influence of Visual Light Detection on Myopic Eye Growth

Visual stimuli have many different characteristics that are detected by the retina and converted into neuronal signals by retinal circuits. The properties of light that have been shown to influence refractive development and/or susceptibility to myopia include luminance, wavelength, temporal frequency, and contrast (ON/OFF stimuli) (for a review, see Muralidharan et al. 2021). The first step in vision is the capture of photons of light by two types of retinal photosensors: conventional rod and cone photoreceptors found in the outer retina and ayptical opsins (e.g., Opn4 and Opn5) located in the inner retina and anterior structures. It is unclear whether the third atypical opsin, Opn3, truly acts as a mammalian photopigment, despite its expression in ocular tissues (Linne et al. 2023). These photoreceptors and photopigments are stimulated by different luminance properties and wavelengths, providing insight into how different visual stimuli activate different retinal pathways.

#### 3.2.1. Effects of the light cycle on refractive development.

Experiments using WTC57BL/6 mouse models of myopia have tested multiple aspects of visual stimuli. First, the balance of light and dark periods has been shown to be important for refractive development. A longer photoperiod [18:6 hours (light:dark) versus 8:18] resulted in myopic shifts with reduced retinal *Egr1* and scleral collagen fiber diameters (Zhou et al. 2010a). Similarly, mice housed in constant light became more myopic and had larger myopic shifts with FDM compared to mice housed in constant darkness, which became hyperopic and did not develop myopic shifts (Tkatchenko et al. 2013). Furthermore, mice exposed to flickering light for the light phase (12 h at 2 Hz, 250 lux) developed more myopia than mice exposed to normal visual conditions but less myopia than mice exposed to form deprivation (Yu et al. 2011). Daily variations in ocular dimensions suggest that circadian rhythms could play a role in refractive development, and a retina-specific knockout of *Bmal1*, a core molecular clock gene, led to more negative refractions and increased ALs compared to WT (Stone et al. 2019). Collectively, these experiments suggest that longer or more frequent exposure (flicker) to mesopic light promotes myopic eye growth and that circadian rhythms may be important for normal refractive development.

#### 3.2.2. Refractive development is influenced by a range of luminance.

Bright light has been shown to be inhibitory for myopia development in children and animal models (for a review, see Muralidharan et al. 2021). Similarly, C57BL/6 mice exposed to bright light (2,500–5,000 lux) had less FDM compared to mice exposed to normal laboratory lighting (100–200 lux) (Chen et al. 2017). To investigate the effects of a broader range of ambient luminance, Landis et al. (2021) exposed mice to scotopic (0.0016 cd/m^2^), mesopic (16 cd/m^2^), or photopic (4,700 cd/m^2^) lighting on a 12:12-hour cycle to target rod, mixed rod and cone, or cone-only retinal pathways, respectively. The resulting data revealed that mice in both scotopic and photopic lighting developed less LIM than mice exposed to mesopic lighting. Both studies indicated a role for dopamine in this process. In the study by Chen et al. (2017), a dopamine D1 receptor antagonist reversed the inhibitory effects of bright light exposure. In the study by Landis et al. (2021), the retinas from the bright, photopic group had the highest dopamine turnover (DOPAC-to-dopamine ratio). Thus, photopic lighting may cause increased dopamine release. The mechanism causing the reduced incidence of myopia with scotopic lighting was not identified but could also be related to dopamine, since ON pathway stimulation, such as that driven by rod activation of rod ON bipolar cells, causes increased release of dopamine (Perez-Fernandez et al. 2019).

#### 3.2.3. Investigating the role of rod and cone pathways in refractive growth.

Using the power of the mouse model, experiments can also be performed to investigate how targeting specific retinal neurons or pathways involved in processing of visual stimuli alters refractive development and the response to myopigenic stimuli. When rod photoreceptor function was eliminated due to a rod transducin-α mutation (*Gnat1*), mice had abnormal refractive development in which their eyes were more hyperopic at young ages compared to WT mice. In addition, *Gnat1*^−/−^ mice did not develop a myopic shift in response to form deprivation, even after prolonged exposure (Park et al. 2014). These experiments indicate that functional rod photoreceptors are necessary for the response to form deprivation. In contrast, when cone photoreceptor function was blocked using a cone transducin-α mutation (*Gnat2*), mice had normal refractive development and increased susceptibility to form deprivation compared to WT mice (Chakraborty et al. 2019). It appears that both rod and cone pathways play important roles in refractive and myopic development.

#### 3.2.4. Influence of spectral properties of light on refractive error.

Investigators have taken special interest in the spectral properties of visual stimuli due to the possible influence of longitudinal chromatic aberration as an optical cue to signal the direction of eye growth with defocus. Specific wavelengths of light, such as short (blue, violet) or long (red) wavelengths, have been shown to increase or decrease susceptibility to myopia depending on the model species (for a review, see Troilo et al. 2019). The mouse model has been particularly useful to explore the underlying mechanisms of spectral stimuli on eye growth. For instance, Strickland et al. (2020) reported that mice housed in violet light (400 nm), compared to those housed in white (420–680 nm) and green (525 nm) light, had greater hyperopia during refractive development. Furthermore, mice housed in violet light developed smaller myopic shifts with LIM. Both effects were reversed when functional cones were not present in the retina (*Gnat2*^−/−^ mice), suggesting that retinal cone pathways were needed for this protective effect. Additionally, increasing violet light (360–400 nm) transmission prior to lights-off resulted in less myopic shift and choroidal thinning with LIM (Jeong et al. 2023). Furthermore, even a 70% reduction of violet light transmission, modeling potential attenuation of violet light through the ocular lens, provided significant benefits. Ji et al. (2021) used the mouse model to provide selective increased or decreased stimulation of middle-wavelength cones by housing the mice in green light (460–600 nm), which caused myopia after 12 weeks, as compared to a genetic knockout of M opsin (*Opn1mw*^−/−^), which induced hyperopia and had less dopamine than WT mice in white light. However, it should be noted that this green light exposure was found to induce retinal degeneration by eight weeks, creating a confounder in the interpretation of the data (Ji et al. 2022). As noted in Section 3.1, clinical retinal diseases with photoreceptor degeneration are associated with both myopia and hyperopia.

#### 3.2.5. Atypical opsin pathways modulate refractive development and myopia.

Atypical opsins are an intriguing target for the modulation of refractive eye growth, since they are luminance detectors found in RGCs, modulate circadian rhythms, and play a role in dopamine release (Contreras et al. 2021, Guido et al. 2022). Two papers published in 2022 used different methods to genetically disrupt Opn4 signaling, and both found a strong modulatory effect of melanopsin on refractive development (Chakraborty et al. 2022, Liu et al. 2022). When *Opn4* was eliminated using *lacZ* β-galactosidase (*Opn4*^−/−^) (Chakraborty et al. 2022) or by replacement of *Opn4* with *Cre* recombinase (*Opn4*^*Cre/Cre*^) (Liu et al. 2022), melanopsin RGCs were still intact and could still receive information from conventional photoreceptors but could not be directly activated with light. The loss of melanopsin resulted in shorter ALs and hyperopic refractions in both models, although *Opn4*^−/−^ mice had a steeper refractive development curve with more myopia at young ages and more hyperopia at older ages compared to WT mice (Chakraborty et al. 2022). The methodology to inactivate *Opn4* altered the response to form deprivation with *lacZ* replacement resulting in greater FDM (Chakraborty et al. 2022), and *Opn4*^*Cre/Cre*^ causing reduced FDM (Liu et al. 2022). When *Opn4* was targeted with diphtheria toxin A (DTA) (Chakraborty et al. 2022), melanopsinsaporin (MEL-SAP), or AAV-CAG-DIO-DTA injected into *Opn4*^*Cre/Cre*^ mice (*Opn4*^*Cre/Cre*^-DTA) (Liu et al. 2022), the RGCs were ablated, eliminating both conventional input and direct photosensing. Absence of melanopsin RGCs resulted in mice with shorter ALs and either more hyperopic (Chakraborty et al. 2022) or more myopic (Liu et al. 2022) refractions. *Opn4*^*Cre/Cre*^-DTA mice also had a shorter corneal radius of curvature. The response to FDM in these animals was also dependent on methodology, with *Opn4*^*DTA*^ mice developing increased FDM,*Opn4*-MEL-SAP mice developing decreased FDM, and *Opn4*^*Cre/Cre*^-DTA mice having an unchanged myopic shift. Interestingly, dopamine and DOPAC levels were significantly higher in *Opn4*^*DTA*^ mice, perhaps explaining the increased hyperopia with normal growth. With FDM, dopamine and DOPAC were lower in *Opn4*^−/−^ mice, and L-DOPA treatment partially inhibited the myopic shift with form deprivation, suggesting a potential role of dopamine in the response (Chakraborty et al. 2022). Finally, when intrinsically photosensitive RGCs (ipRGCs) were chemogenetically activated with hM3Dq and housed in the dark, the mice became more hyperopic than controls, with a larger corneal radius of curvature and longer ALs (Liu et al. 2022). In summary, these studies suggest that elimination of melanopsin or ipRGCs caused decreased AL and modulation of refractive development, while activation of ipRGCs increased AL and hyperopia. Furthermore, these studies suggest a role for ipRGCs in the development of FDM and a contribution by them to corneal curvature development.

The other two atypical opsins, Opn5 and Opn3, have also been recently studied. The protective effects of violet light discussed above have been attributed to Opn5, which is found in a subset of RGCs. When*Opn5*^−/−^ mice were exposed to violet light at dusk (ZT9–12), the protective effects of violet light on refractive error, AL, and choroidal thickness after LIM were abolished (Jiang et al. 2021). Additionally, Opn3, which is sensitive to blue light in nonmammalian species and widely expressed in the body and eye, was also implicated in refractive eye growth (Linne et al. 2023). *Opn3*^−/−^ mice had more myopic refractions during development and decreased lens thickness, shallower aqueous chamber depth, and shorter AL. Linne et al. (2023) created a retina-specific *Opn3* knockout and found that the mice did not have myopia, suggesting that germline *Opn3*, and not retinal *Opn3*, is responsible for the phenotype observed in *Opn3*^−/−^ mice. The data also showed that both germline and retina-specific *Opn3* null mice had similar responses to LIM (Linne et al. 2023). Overall, these authors concluded that nonretinal *Opn3* influences refractive development and the response to LIM. Collectively, these experiments with atypical opsins demonstrated that numerous transgenic approaches can be used to manipulate genes, cell types, and/or pathways. The results show that Opn3, Opn4, and Opn5 signaling modulate refractive development through complex mechanisms.

#### 3.2.6. Influence of ON and OFF retinal pathways in refractive eye growth.

Photoreceptors comprise the first step in the visual pathway, followed by synapses with ON and OFF bipolar cells that form the basis for the parallel ON and OFF retinal pathways that are activated by light or dark stimuli. As noted above, retinal diseases, such as CSNB, which is caused by mutations in the ON pathway, are also highly associated with myopia. In fact, individuals with bipolar cell–related retinal dystrophies have the greatest risk for high myopia (Hendriks et al. 2017). Several experiments have been performed to evaluate the influence of retinal ON and OFF pathways on myopia using mouse models. Mice with ON pathway defects that are caused by mutations in the postsynaptic side of the photoreceptor to ON bipolar cell synapse (Pardue & Peachey 2014) have all shown increased susceptibility to induced myopia (FDM or LIM) (Chakraborty et al. 2015, Pardue et al. 2008, Wilmet et al. 2022). With normal development, *Nyx*^−/−^ mice show more hyperopia at early ages than WT (Pardue et al. 2008), while *mGluR6*^−/−^ mice were more myopic (Chakraborty et al. 2015), and *Gpr179*^−/−^ mice were similar to WT mice (Wilmet et al. 2022). All of these ON-pathway mutants had reduced dopamine and/or DOPAC levels. In comparison, *Vsx1*^−/−^ mice were evaluated as an OFF-pathway mutant. *Vsx1* is found on only a subset of OFF cone bipolar cells (types 2, 3, and 4) (Chow et al. 2001, 2004; Shi et al. 2011). *Vsx1*^−/−^ mice had normal refractive development compared to WT mice and a similar response to FDM (Chakraborty et al. 2014). There are known differences in refractive error and eye size across mouse strains (Tkatchenko et al. 2019, Zhou & Williams 1999), and the *Vsx1*^−/−^ mice were from a 129S1/Sv background, a mouse strain that has minimal response to FDM.

Collectively, these experiments support the evidence that visual stimuli influence refractive development and susceptibility to myopia, with bright and dim lighting being protective and mesopic lighting being myopigenic. Furthermore, mouse models provide insight into potential retinal cell types and pathways that might be involved, including rods, cones, atypical opsins, and retinal ON pathways. There is good agreement across the studies that more hyperopia is associated with increased retinal dopamine signaling, and more myopic refractions are associated with decreased retinal dopamine signaling.

### 3.3. Retinoscleral Signaling in Myopic Eye Growth

A major gap in our knowledge of what sustains myopia is the signaling cascade that regulates refractive eye growth. This cascade is thought to translate visual cues into signals that regulate axial elongation through a retina-to-sclera pathway (for a review, see Brown et al. 2022b). Work in the mouse model of myopia has focused on tissue-specific changes of a few key molecules: mainly dopamine changes in the retina and retinoic acid changes affecting the sclera.

#### 3.3.1. Dopaminergic signaling and other retinal candidates.

While experiments in other species have shown decreased levels of retinal dopamine with FDM and LIM, the results have been mixed with mice. Using high-performance liquid chromatography (HPLC), dopamine levels and dopamine-associated proteins were not changed with FDM in C57BL/6 mice (Wu et al. 2015). However, C57BL/6 mice are known to be deficient in melatonin, and testing CBA/CaJ mice with proficient melatonin showed a decrease in dopamine with FDM (Qian et al. 2022). Furthermore, the contributions of dopamine to myopic eye growth have been confirmed in mice by using L-DOPA treatment to slow myopic eye growth or transgenic mice with increased or decreased levels of VMAT2 (the vesicular packing protein for dopamine) to reduce or enhance myopic eye growth, respectively (Landis et al. 2020). Eliminating tyrosine hydroxylase from the retina caused more myopic refractions and steeper corneas; however, the response to FDM was similar to that of WT controls (Bergen et al. 2016). Reducing dopamine levels with 6-hydroxydopamine results in myopia with shorter ALs and increased susceptibility to FDM with longer ALs (Wu et al. 2016). Dopamine receptors have also been targeted using pharmacology or genetics. Apomorphine, a nonspecific dopamine agonist, attenuates FDM in mice (Yan et al. 2015). D2 antagonists reduced the response to FDM (Huang et al. 2014, 2018), while the ability of D2 agonists to modulate the response to FDM was dependent on the concentration (Huang et al. 2020). Using tissue-specific knockouts, Huang et al. (2022) showed that D2 receptors in the retina are important for dopaminergic regulation of FDM, but D2 receptors in scleral fibroblasts are not.

Dopamine may not be the only signaling molecule for myopic eye growth. The levels of GABA transporters increased in the LIM retina and were reduced after atropine treatment (Barathi et al. 2014). Atropine is a muscarinic antagonist, but other functions are possible. Deletion of the muscarinic cholinergic receptor gene (*Chrm2*) resulted in higher collagen I and lower collagen V levels in the sclera and reduced myopic shift with LIM (Barathi et al. 2013). Moreover, pharmacologically blocking M2 muscarinic receptors was protective for experimental myopia in the mouse (Barathi et al. 2013). Furthermore, knockdown of *Egr1*, which encodes a transcription factor, also known as ZENK, that has been shown to be reduced with myopia development, caused increased AL and a myopic shift (Schippert et al. 2007). Crocetin, a drug shown to modulate EGR1 levels, attenuates LIM (Mori et al. 2019).

#### 3.3.2. Potential retinal pigment epithelial or scleral signaling candidates and other molecules.

The signaling for refractive eye growth needs to transverse the retina, RPE, and choroid to reach the sclera for collagen remodeling and axial elongation. Thus, it is likely going to involve a signaling cascade with multiple molecules. Mouse studies in the retina have provided the following insights into possible candidates.

As in other species, all-*trans* retinoic acid (atRA) increased with FDM (Brown et al. 2022a). Feeding mice atRA resulted in increased AL, myopic refractions, decreased scleral stiffness, and increased scleral permeability (Brown et al. 2023), similar to the effects from FDM (Brown et al. 2022a). One potential binding partner of atRA is interphotoreceptor retinoid-binding protein (IRBP), which leads to highly myopic eyes from birth when the *Irbp* gene is knocked out (Markand et al. 2016). Additionally, other transcription factors, like sonic hedgehog (Shh), which requires the interaction of other factors such as atRA, Pax6, and fibroblast growth factors, may play a role. *Shh* transcript levels are increased with FDM, and administration of Shh-N stimulated the development of myopia, while inhibiting the Shh pathway with cyclopamine reduced FDM and axial elongation (Qian et al. 2009).

Multiple other genes and treatments have been shown to alter collagen and MMP2 expression (Table 1). For instance, as described in Section 3.1, HIF-1α signaling and inflammatory signals could play a role. Hypoxia may reduce choroidal blood flow and cause scleral hypoxia (Zhao et al. 2020b). Furthermore, drugs inhibiting ER stress have been shown to reduce scleral collagen fiber thinning in LIM mice (Ikeda et al. 2022). However, these pathways are ubiquitous in multiple tissues, so it can be difficult to know if these are primary or secondary effects, since scleral remodeling is the final step of myopia development.

## 4. THE POWER OF MOUSE MODELS FOR MYOPIA RESEARCH

The use of the mouse model for myopia studies has significantly increased, a necessary step to uncovering mechanisms underlying myopia development. Nonmouse models have laid a robust foundation for understanding pathways and environments associated with myopia (Troilo et al. 2019) and contributed greatly to investigations in mice. However, by leveraging genetic techniques with the combination of environmental and pharmacological treatments, specific genes, cells, and pathways can be targeted using multiple different manipulations. Moreover, genetic deletion and rescue, as well as gain- and loss-of-function experiments, can provide evidence of essential cells or pathways involved in myopia development. Additionally, the mouse model allows researchers to directly test myopia-associated genes derived from humans. As utilization of the powerful genetic and experimental tools in the mouse model continues to increase, the mouse is poised to significantly change our understanding of myopia mechanisms. Despite some challenges with the mouse model (e.g., small eyes, low visual acuity), their visual system is surprisingly similar to that of the peripheral human retina, which makes up the vast majority of retinal visual signaling and is thought to be critical for myopia development. Therefore, mechanistic knowledge of myopigenesis in mice can be translated to the human condition and guide the optimization of current treatments and development of novel evidence-based interventions to prevent or slow myopia progression.

## Figures and Tables

**Table 1:** Studies conducted in the mouse model investigating the effect of different factors on refractive development

Citation	Manipulation	Model	Measure
**Syndromes and diseases associated with myopia**			
Pardue et al. 2008	Gene: *Nyx*	Normal development and FDM	**Refractive error** **
Wisard et al. 2011	Gene: *Irbp*	Normal development	Refractive error**, **axial length, ocular axial parameters, equatorial diameter**^a^, **eye weight**
Park et al. 2013	Gene: *Pde6b*	Normal development and FDM	Refractive error**, **axial length**
Tekin et al. 2013	Gene: *Slitrk6*	Normal development	**Axial length**^b^,^c^, ocular axial parameters^b^,^c^
Aikio et al. 2013	Gene: *Col18a1*	Normal development	**Axial length**^d^, **ocular axial parameters, eye volume**^d^, **eye weight**
Storm et al. 2014	Gene: *Lrp2*	Normal development	**Axial length**^b^, **ocular axial parameters**^b^
Ma et al. 2014	Drugs modulating Wnt signaling	Normal development, FDM, and lid suture	Refractive error*^e^, **axial length**^f^,^g^
Liu et al. 2015	Gene: *Apc*	Normal development	Refractive error*, **axial length,** corneal curvature, **ocular axial parameters**
Markand et al. 2016	Gene: *Irbp*	Normal development and FDM	Refractive error**, **axial length,** corneal curvature, **ocular axial parameters, eye weight, equatorial diameter**^a^
Zhu et al. 2021	Gene: *Irbp*	Normal development and LIM −25 D	Refractive error***, **axial length**^c^, **lens cross-sectional area**^c^
Liu et al. 2021	Gene: *Apc* Drug: niclosamide	Normal development and FDM	Refractive error*, **axial length,** corneal curvature, **ocular axial parameters**
Mai et al. 2022	Genes: *Lrp2, Srebp2, Bmp2*	Normal development	**Axial length, ocular axial parameters, equatorial diameter** ^f^
Wilmet et al. 2022	Gene: *Gpr179*	Normal development and LIM −25 D	**Refractive error** *
Zhang et al. 2022	Genes: *Lrp2, Vegf, Vhl*	Normal development	Refractive error**, **axial length, ocular axial parameters, choroidal thickness**
**Genetic risk factors of myopia**			
Hysi et al. 2010	Gene: *Rasgfr1*	Normal development	Axial length^f^, equatorial diameter^f^, eye weight, **lens weight**
Nair et al. 2011	Gene: *Prss56*	Normal development	**Axial length**^a^, **ocular axial parameters**^f^, **equatorial diameter**^a^, lens diameter^a^, **choroidal thickness**^b^
Tkatchenko et al. 2015	Gene: *Aplp2*	Normal development and FDM	**Refractive error** *
Piekutowska-Abramczuk et al. 2016	Gene: *Sco2*	Normal development	Axial length^g^
Paylakhi et al. 2018	Genes: *Prss56*, *Egr1*	Normal development	Refractive error*, **axial length, ocular axial parameters, equatorial diameter**^a^
Wu et al. 2018	Drugs: eIF2 and mTOR signaling	Normal development and FDM	Refractive error***, **axial length, ocular axial parameters**
Szczerkowska et al. 2019	Gene: *Zfp644*	Normal development	**Axial length**^g^, **ocular axial parameters**^g^, **lens diameter**^g^
Zhao et al. 2020b	Gene: *Hif1a*	Normal development and FDM	Refractive error***, **axial length,** ocular axial parameters
Koli et al. 2021	Genes: *Prss56*, *Mfrp*, *Adamts19, Irbp, Egr1*	Normal development	**Axial length, ocular axial parameters**
Wu et al. 2022	Gene: *Hif2a*	Normal development and FDM	Refractive error*, axial length, ocular axial parameters
Zhao et al. 2022	Gene: *Vipr2* Drug: VIPR2 antagonist	Normal development and FDM	Refractive error***, **axial length, ocular axial parameters**
Liang et al. 2022	miR-328–3p Drug: atropine	FDM	**Axial length** ^f^
Jiao et al. 2023	Gene: *Vdr* Drug: calcipotriol	Normal development and FDM	Refractive error*, **axial length, ocular axial parameters**
Tian et al. 2023	Gene: *Glra2*	Normal development	Refractive error*, axial length, corneal curvature^h^, **ocular axial parameters**
J. Chen et al. 2023	Gene: *Psmd3*	Normal development	**Axial length**
**Atropine treatment**			
Barathi & Beuerman 2011	Drug: atropine	LIM −10 D	Refractive error*, **axial length, corneal curvature, ocular axial parameters**
Barathi et al. 2013	Genes: *Chrm1–5* Drugs: muscarinic antagonists	Normal development, LIM −15 D, and FDM	Refractive error*, **axial length**
Barathi et al. 2014	Drug: atropine	Normal development and LIM −15 D	Refractive error*, **axial length**
Jiang et al. 2018	Drug: atropine	LIM −30 D	Refractive error**, **axial length**, corneal curvature, **ocular axial parameters**
Lin et al. 2021	Drugs: atropine and drug screen	Normal development and FDM	Refractive error**^e^, **axial length**^g^
van der Sande et al. 2023	Gene: *Lrp2* Drug: atropine	Normal development	**Axial length, ocular axial parameters**
**Extracellular matrix remodeling of the sclera**			
Chakravarti et al. 2003	Genes: Lum, *Fmod*	Normal development	**Axial length** ^f^
Zhou et al. 2010b	Gene: *Adora2a*	Normal development	Refractive error*, **axial length**, corneal curvature, **ocular axial parameters**
Song et al. 2016	Gene: *Lum*	Normal development	**Axial length** ^f^
Zhao et al. 2018	Gene: *Mmp2*	Normal development and FDM	Refractive error***, **axial length**, corneal curvature^h^, **ocular axial parameters**
Zhao et al. 2020a	Genes: *Mmp2, Ccl2*	Normal development and FDM	Refractive error*, **axial length**, ocular axial parameters
Ikeda et al. 2020	Drug: lactoferrin	LIM −30 D	Refractive error**, **axial length**
Zhao et al. 2021	Gene: *Pde4b*	Normal development	Refractive error*, axial length, corneal curvature^h^, **ocular axial parameters**
Pan et al. 2021	Drugs: DHA and EPA	FDM	Refractive error*, axial length, ocular axial parameters
Mori et al. 2022	Gene: *fat-1* Drugs: PUFAs	LIM −30 D	Refractive error**, **axial length, choroidal thickness**
Ikeda et al. 2022	Genes: *Perk, Atf6* Drug: oxidative stress modulation	Normal development and LIM −30 D	Refractive error**, **axial length, ocular axial parameters**
Z. Chen et al. 2023	Gene: *Nlrp3*	Normal development and FDM	Refractive error*^i^, axial length^a^
Hou et al. 2023	Drug: ginkgo biloba extract	LIM −30 D	Refractive error**, **axial length, choroidal thickness**
Liang et al. 2023	Drugs: lactoferrin and derivatives	LIM −30 D	Refractive error**, **axial length, choroidal thickness**
**Ocular or visual light detection**			
Zhou et al. 2010a	Environment: 12:12 h, 18:6 h, and 6:18 h LD cycles	Normal development	Refractive error*, **axial length, corneal curvature, ocular axial parameters**
Yu et al. 2011	Environment: flickering light	Normal development	Refractive error*, **axial length**^g^
Tkatchenko et al. 2013	Environment: 12:12 h LD cycle, LL, DD	Normal development and FDM	Refractive error*, **axial length**^c^, **ocular axial parameters**^c^ equatorial diameter^c^, **eye circumference**^c^, corneal curvature^c^
Park et al. 2014	Gene: *Gnat1*	Normal development and FDM	Refractive error**, axial length
Chakraborty et al. 2014	Gene: *Vsx1*	Normal development and FDM	Refractive error**, axial length, corneal curvature
Chakraborty et al. 2015	Gene: *mGlur6*	Normal development and FDM	Refractive error**, axial length, corneal curvature
Chen et al. 2017	Environment: bright light Drug: D1R antagonist	Normal development and FDM	Refractive error*, **axial length, ocular axial parameters**
Chakraborty et al. 2019	Gene: *Gnat2*	Normal development and FDM	Refractive error**, axial length, corneal curvature
Stone et al. 2019	Gene: *Bmal1*	Normal development	Refractive error**, **axial length,** corneal curvature, **ocular axial parameters**
**Ocular or visual light detection**			
Strickland et al. 2020	Gene: *Gnat2* Environment: white, green, and violet light	Normal development and LIM −10 D	Refractive error**, axial length, corneal curvature, ocular axial parameters
Ji et al. 2021	Gene: *Opn1mw* Environment: white and green light	Normal development	Refractive error*, **axial length, ocular axial parameters**
Jiang et al. 2021	Gene: *Opn5* Environment: violet, red, green, and blue light	Normal development and LIM −30 D	Refractive error**, **axial length, choroidal thickness**
Landis et al. 2021	Environment: scotopic, mesopic, and photopic light	Normal development and LIM −10 D	Refractive error**, axial length, corneal curvature, ocular axial parameters
Chakraborty et al. 2022	Gene: *Opn4*	Normal development and FDM	Refractive error**, **axial length,** corneal curvature, **ocular axial parameters**
Liu et al. 2022	Gene: *Opn4; rd/rd* cl Environment: LL, DD	Normal development and FDM	Refractive error*, **axial length, corneal curvature, ocular axial parameters**
Ji et al. 2022	Environment: green light	Normal development	Refractive error*, **axial length**^g^
Jeong et al. 2023	Environment: violet light	LIM −30 D	Refractive error**, **axial length, choroidal thickness**
Linne et al. 2023	Gene: *Opn3*	Normal development and LIM −30 D	Refractive error**, **axial length, ocular axial parameters,** choroidal thickness
**Retinoscleral signaling**			
Schippert et al. 2007	Gene: *Egr1*	Normal development	Refractive error*, **axial length, corneal curvature, ocular axial parameters**
Qian et al. 2009	Drug: Shh-N or cyclopamine	Normal development and FDM	Refractive error*^e^, **axial length**^f^
Huang et al. 2014	Gene: *Drd2* Drug: sulpiride	Normal development and FDM	Refractive error*, **axial length,** corneal curvature, **ocular axial parameters**
Yan et al. 2015	Drug: apomorphine	Normal development and FDM	Refractive error*, **axial length,** corneal curvature^h^, **ocular axial parameters**
Wu et al. 2016	Drug: 6-hydroxydopamine	Normal development and FDM	Refractive error*, **axial length, corneal curvature, ocular axial parameters**
Bergen et al. 2016	Gene: *Th*	Normal development and FDM	Refractive error**, **axial length, corneal curvature, ocular axial parameters**
Huang et al. 2018	Gene: *Drd1, Drd2* Drug: apomorphine	Normal development and FDM	Refractive error*, **axial length,** corneal curvature^h^, **ocular axial parameters**
Mori et al. 2019	Drug: crocetin	LIM −30 D	Refractive error**, **axial length,** corneal curvature, **choroidal thickness**
Huang et al. 2020	Gene: *Drd2* Drugs: quinpirole, aripiprazole	Normal development and FDM	Refractive error*, **axial length**, corneal curvature^h^, **ocular axial parameters**
Landis et al. 2020	Gene: *Vmat2* Drug: L-DOPA	Normal development and FDM	Refractive error**, axial length, corneal curvature, ocular axial parameters
**Retinoscleral signaling**			
Huang et al. 2022	Gene: *Drd2* Drug: sulpiride	Normal development and FDM	Refractive error*, **axial length,** corneal curvature^h^, **ocular axial parameters**
Qian et al. 2022	Drug: luzindole	Normal development and FDM	Refractive error*, **axial length,** corneal curvature, ocular axial parameters
Brown et al. 2023	Drug: atRA	Normal development	Refractive error**, axial length, corneal curvature, **ocular axial parameters**

This table highlights studies investigating the effect of genetic, pharmacological, and environmental manipulations on refractive development under normal visual conditions or with experimental myopia (FDM or LIM). Ocular axial parameters include any axial parameter in addition to axial length, e.g., corneal thickness, anterior chamber depth, lens thickness, vitreous chamber depth, or retinal thickness. Standard methods for measurements were defined as photorefraction (for refractive error), OCT or similar (for axial length, choroidal thickness, and ocular axial parameters), keratometer (for corneal curvature), and weighing (for eye weight and lens weight). The state of mice during refractive error measurements is given by asterisks:

* indicates awake,

** indicates asleep, and

*** indicates that the state is not explicitly stated. Bold text indicates measurements that were reported as statistically different between the control and intervention group in any of the comparisons. Nonstandard methods are indicated by footnotes. Several articles could be classified in multiple subsections but were included only in one.

a Measurement with calipers.

b Histology.

c Magnetic resonance imaging.

d No clear information.

e Streak retinoscopy.

f Photography of enucleated globes.

g Ultrasonography.

h Corneal curvature calculation based on OCT.

i Direct ophthalmoscopy.

Abbreviations: D, diopters; DD, constant darkness; FDM, form deprivation myopia; LD, light–dark; LIM, lens-induced myopia; LL, constant light; OCT, optical coherence tomography.

## SUMMARY POINTS

1. Myopia is a global visual health concern that is rapidly increasing in prevalence and requires more effective clinical interventions that prevent myopia progression.
2. Mice show features of human refractive and myopic development and have similar ocular and visual properties as humans.
3. The mouse model of myopia fills a gap in mechanistic investigation into the cause and progression of myopia.
4. Detailed mechanistic studies in mice, utilizing a combination of genetic, environmental, and pharmacological manipulations, provide compelling evidence for underlying mechanisms of myopia that are poised to optimize clinical interventions.
5. Genes associated with myopia in humans are testable in mice and have revealed similar effects on refractive development and myopigenesis through multiple pathways.
6. Mouse models of myopia provide insight into potential retinal cell types and retinoscleral signaling pathways that are important in different visual environments for refractive development and myopia.
7. Results from mouse studies illustrate the complexity of ocular features that can influence refractive power.
8. Given the experimental tools available and the capability to combine different approaches, the mouse has the potential to significantly shape our understanding of myopigenesis.

## FUTURE ISSUES

1. Pursuing investigations that combine genetic, environmental, and pharmacological manipulations in myopic mice may offer novel insights into causal mechanisms of myopia.
2. More studies leveraging genetic manipulations targeting loss and gain of function in cell- and tissue-specific pathways are needed to address the gap in fully understanding myopia mechanisms.
3. Several unknown mechanisms of action of myopia interventions, such as atropine, can be solved in the mouse by utilizing genetic experimental tools.
4. Replication of less well-studied genes and pathways will increase evidence regarding their potential role in myopia development.
5. Further optimization of methods to measure refractive eye growth in mice, such as refractive error and axial length, will be critical to improve measurements in the small mouse eye.
